# Methods and representativeness of a European survey in children and adolescents: the KIDSCREEN study

**DOI:** 10.1186/1471-2458-7-182

**Published:** 2007-07-26

**Authors:** Silvina Berra, Ulrike Ravens-Sieberer, Michael Erhart, Cristian Tebé, Corinna Bisegger, Wolfgang Duer, Ursula von Rueden, Michael Herdman, Jordi Alonso, Luis Rajmil, the European KIDSCREEN group [kidscreen]

**Affiliations:** 1 Agency for Quality, Research and Assessment in Health (AQuRA Health, formerly Catalan Agency for Health Technology Assessment and Research). Barcelona, Spain; 2 School of Public Health, WHO Collaborating Center for Child and Adolescent Health Promotion; University of Bielefeld, Germany; 3 Department of Social and Preventive Medicine, University of Berne, Berne, Switzerland; 4 Ludwig Boltzmann-Institute for Sociology of Health and Medicine, University of Vienna, Vienna, Austria; 5 Institut Municipal d'Investigació Mèdica, Barcelona, Spain

## Abstract

**Background:**

The objective of the present study was to compare three different sampling and questionnaire administration methods used in the international KIDSCREEN study in terms of participation, response rates, and external validity.

**Methods:**

Children and adolescents aged 8–18 years were surveyed in 13 European countries using either telephone sampling and mail administration, random sampling of school listings followed by classroom or mail administration, or multistage random sampling of communities and households with self-administration of the survey materials at home. Cooperation, completion, and response rates were compared across countries and survey methods. Data on non-respondents was collected in 8 countries. The population fraction (PF, respondents in each sex-age, or educational level category, divided by the population in the same category from Eurostat census data) and population fraction ratio (PFR, ratio of PF) and their corresponding 95% confidence intervals were used to analyze differences by country between the KIDSCREEN samples and a reference Eurostat population.

**Results:**

Response rates by country ranged from 18.9% to 91.2%. Response rates were highest in the school-based surveys (69.0%–91.2%). Sample proportions by age and gender were similar to the reference Eurostat population in most countries, although boys and adolescents were slightly underrepresented (PFR <1). Parents in lower educational categories were less likely to participate (PFR <1 in 5 countries). Parents in higher educational categories were overrepresented when the school and household sampling strategies were used (PFR = 1.78–2.97).

**Conclusion:**

School-based sampling achieved the highest overall response rates but also produced slightly more biased samples than the other methods. The results suggest that the samples were sufficiently representative to provide reference population values for the KIDSCREEN instrument.

## Background

Health-related quality of life (HRQoL) measurement can provide useful data for health services research. The measurement of HRQoL is currently more advanced in adult than in child populations, though a substantial number of generic and disease-specific questionnaires now exist to measure HRQOL in younger respondents[1].

Reference values can facilitate the interpretation of scores on an instrument by providing a point of comparison for individual or group responses obtained in clinical or epidemiological studies [2]. This is particularly helpful if reference values are available by age group, sex, and other relevant characteristics. Population norms can also be used to develop norm-based, standardized scores. In this case, scores are standardized so that a score of 50, for example, represents the mean score for the general population in a given country or region, and 10 points is equivalent to 1 standard deviation[3]. Reference values and norm-based, standardized scores can be used to determine the extent to which an individual or group differ from the standard for their age, sex, etc.

General population norms are available for some generic HRQoL questionnaires for adults [4], but they are not usually developed simultaneously in different countries. Likewise, reference values are not available for most of the instruments designed for use in children and adolescents. When obtaining reference values, the sampling techniques used should ensure representativeness in terms of age, socioeconomic status, and gender and any other variables considered important [5]. Methods which systematically exclude certain segments of the population should be avoided and the external validity of the results should be evaluated[6] by comparison with an external source of information such as those provided by census data. Previous studies have indicated that the social and individual characteristics of eligible subjects, sampling, and fieldwork procedures can influence the decision to cooperate or not in a survey[7,8] and it has been found that non-response tends to be higher among individuals with lower educational levels and in low-income groups [9,10].

The KIDSCREEN is a generic questionnaire designed to measure HRQoL in children and adolescents aged 8 to 18. The instrument was developed and pilot tested in seven European countries [11], and is intended primarily for use in multinational clinical trials and health interview surveys [12,13]. Three different versions of the instrument have been developed: the core version contains 52 items spread over 10 dimensions [11], the second has 27 items in 5 dimensions, and a 10 item version exists which provides a single index score [12]. Versions for both children and parents or guardians are available for all variants of the questionnaire. The psychometric properties of the 52-item version were tested after administration of the instrument to respondents in 13 European countries [13]. A further aim of the KIDSCREEN project was to obtain reference values or norms which would facilitate interpretation of scores with the instrument.

The objective of the present analysis was to compare the results obtained using different sampling and questionnaire administration methods in the international KIDSCREEN project in terms of participation, response rates, and completion rates. A further objective was to assess the representativeness of the samples obtained in the thirteen participating countries by examining their external validity.

## Methods

### Participating countries

The following countries participated in the KIDSCREEN study: Austria (AT), Czech Republic (CZ), France (FR), Germany (DE), Greece (EL), Hungary (HU), Ireland (IE), Poland (PL), Spain (ES), Sweden (SE), Switzerland (CH), The Netherlands (NL), and the United Kingdom (UK).

### Subjects

The target population for the KIDSCREEN study was children and adolescents aged 8–18. A sample size of 1,800 children and adolescents per country was considered necessary to detect a minimally important difference of half a standard deviation (SD) in HRQoL scores within each age strata between children with and without special health care needs or a chronic condition. A response rate of approximately 70% was expected, so the initial sample size was set at 2,400 children and adolescents per country. The sample was drawn to take into account distribution of the target population by age, sex, and region.

### Sampling and questionnaire administration strategies

Three approaches to sample selection and administration were used: 1) telephone sampling followed by a mail survey (AT, CH, DE, ES, FR, and NL); 2) school sampling and survey administration during class-time (EL, HU, IE, and SE), or school sampling followed by a mail survey (PL), and; 3) multistage random sampling of communities and households followed by questionnaire self-administration at home (CZ). In the UK, a combination of telephone and school sampling was used. Fieldwork was carried out between April and November 2003 except in IE, where data were collected in 2005.

All procedures were carried out following the data protection requirements of the European Parliament (Directive 95/46/EC of the European Parliament and of the Council of 24 October 1995 on the protection of individuals with regard to the processing of personal data and on the free movement of such data). Each country was required to respect their national ethical and legal requirements for this type of survey and to obtain signed informed consent from participants.

#### Telephone sampling

A stratified sampling strategy was used to include children proportionally by age, sex, and geographical areas within countries, based on census data. Telephone sampling was performed centrally from Germany, and was carried out using a Computer Assisted Telephone Interview (CATI) with random-digital-dialing (RDD). The RDD randomly generated suffix numbers for each geographical area's prefix number until the desired quota was achieved. Interviewers who had received study-specific training called the random numbers generated to identify households with children or adolescents aged 8–18 years. When such a household was identified, the interviewer asked one of the parents if they would be willing to participate together with their child. If the parent agreed to participate, the questionnaire and other study materials were mailed to the requisite address with a stamped, addressed envelope for return of the completed questionnaire. A telephone hotline was used to provide further information about the survey. Two reminders were sent in cases of non-response (after two and five weeks). Due to initially poor response using RDD in NL and UK, additional phone numbers were obtained from a large data services institute in NL (DIDOC), and school sampling was added as an alternative option in the UK.

#### School sampling

In the case of school sampling, sample selection was also designed to take into account the distribution of children by age, sex, and geographical or administrative regions within countries. Sampling frames were public (state) school (EL, IE, PL) or classroom (HU) listings, and schools were randomly selected within each region. A convenience sample of schools was used in SE. Consent to participate was obtained from parents before study questionnaires were administered. Children whose parents provided informed consent to participate completed their questionnaires in school. Questionnaires were collected during a 2nd visit to schools after 3–7 days.

#### Multistage probability sampling

In CZ, communities were randomly selected from all regions of the country. Households within each selected community were then randomly selected using a local telephone directory. Trained interviewers telephoned to identify families with eligible children, provided standard information on the survey, and delivered the questionnaires to households who agreed to participate. They returned after 2–5 days to collect the completed questionnaires.

In countries which used CATI sampling, as well as PL and CZ, a non-response questionnaire was administered to those who declined participation to collect basic sociodemographic information.

### Study variables

Study materials sent to respondents included the KIDSCREEN-52 questionnaire, several additional instruments which would be used to test the validity of the KIDSCREEN, and a series of questions on sociodemographic and health-related characteristics. Sociodemographic and health-related data was collected from both children and parents, and both children and parents completed the relevant versions of the KIDSCREEN-52 questionnaire.

Variables used to evaluate sample representativeness were the child's sex and age (based on data provided by the children), and the educational level of the child's mother and father (provided by parents). The highest educational qualification of both parents was collected and coded according to the International Standard Classification of Education (ISCED) categories [14] as follows: Low, at most lower secondary (ISCED 0–2); Medium, upper secondary (ISCED 3–4), and; High, tertiary (ISCED 5–6). Household location was collected in five categories (big city or suburbs, town or small city, country village, farm, or house in the country).

Where possible, data was collected from parents who refused to participate to be able to compare characteristics of refusals and participants. Variables collected included: parent-reported child health status, parent's self-perceived overall health, marital status, highest educational level achieved (except for PL, where it was replaced by the mother's highest educational level), and household location.

### Analysis

Participation in the study was assessed by analyzing cooperation, completion, and response rates. Cooperation was defined as the willingness of parents to participate with their children in the study and was computed as the number of parents who agreed to participate divided by the number of eligible households contacted. The result was then multiplied by 100. The completion rate was computed as the number of questionnaires completed by children and adolescents divided by the number of families who agreed to participate, and multiplied by 100. Only valid addresses were used as the denominator for the response rate in the mail survey, since it was considered that people who could not receive mail would not have the opportunity to respond. Finally, response rate was computed as the product of the cooperation rate and the completion rate divided by 100.

In order to assess each national survey's representativeness, they were compared with the corresponding reference population in the Eurostat census database [15]. Samples were compared with the reference data in terms of age (of the population aged 8–18 years), sex, and the highest educational level (according to ISCED categories) of women and men with at least one child aged 8–18 in the household. The Eurostat provides comparable data from statistical offices of the participating European countries.

Participants and refusals were compared in terms of child and parent overall health status, parents' martial status, parent's educational level, and household location in selected countries. SE was not included in the analysis of representativeness because the sample was not intended to be representative.

Differences between the sample's observed and expected (based on Eurostat data) proportions by age and sex were tested for using a binomial test. A chi-squared goodness of fit test was used to analyze whether the sample distribution in terms of level of education differed significantly from the distribution in the reference population. An α < 0.01 was considered to be statistically significant.

Population fractions (PF) by age and sex were computed for each country. The PF was defined as the number of children responding in each age-sex group divided by the number of children with the same characteristics according to the Eurostat data. Population fraction ratios (PFR, e.g. ratio of boys' to girls' PF), and their 95% confidence intervals (95%CI) were also estimated for each country (child's PF and PFR were not calculated for SE and EL because only adolescent samples were recruited in these countries). Population fractions and PFRs were also computed for mothers and fathers according to their educational level, using the mid-level as the reference category. A PFR greater than 1 indicates an "excess" of boys in the sample, while an excess of girls is indicated by a PFR lower than 1. Similarly, a PFR greater than 1 indicates an "excess" of parents in the high (or low) educational level compared to the intermediate educational level. IE was not included in this analysis because data were not available on parents' educational level.

## Results

Table 1 shows the socio-demographic characteristics of the samples included in the participating countries. Data from a total of 22,827 respondents were eligible for analysis. Mean age (SD) in the child and adolescent samples was 9.7 (1.1) and 14.4 (1.7) years, respectively. 51.3% of the child sample was female, and 53.8% of adolescents.

**Table 1:** Socio-demographic characteristics of the children and adolescents in the KIDSCREEN sample, by country

Country *	**Total**	**AT **	**CH**	**CZ**	**DE**	**EL**	**ES**	**FR**	**HU**	**IE**	**NL**	**PL**	**SE**	**UK **
**n (valid cases)**	22,827	1,475	1,701	1,592	1,723	1,174	876	1,049	3,237	1,240	1,885	1,715	3,283	1,877
**Children**														
Age range: 8 – 11														
mean age years (SD)	9.7 (1.1)	9.7 (1.1)	9.8 (1.0)	9.6 (1.0)	9.7 (1.1)	-	9.7 (1.1)	9.5 (1.1)	9.5 (1.1)	10.4 (0.7)	9.6 (1.1)	9.9 (1.0)	-	9.5 (1.0)
Female (%)	51.3	53.5	52.4	50.5	50.0	-	46.1	50.3	55.2	52.6	49.3	53.3	-	47.3
Socio-economic status **														
low FAS (%)	20.0	14.4	10.9	49.5	10.1	-	17.7	7.5	26.7	18.2	11.2	35.7	-	11.2
Medium FAS (%)	45.4	49.2	44.7	41.6	46.5	-	47.6	44.4	47.2	48.9	49.0	48.8	-	36.7
high FAS (%)	34.6	36.4	44.4	8.9	43.4	-	34.7	48.1	26.1	32.9	39.9	15.5	-	52.2
**Adolescents**														
Age range: 12 – 18														
mean age years (SD)	14.4 (1.7)	14.5 (1.8)	14.5 (1.8)	14.9 (1.9)	14.6 (1.9)	14.6 (1.7)	14.7 (1.9)	14.6 (1.9)	14.6 (1.8)	14.6 (1.4)	14.6 (1.8)	14.8 (1.9)	13.7 (1.0)	14.1 (1.6)
Female (%)	53.8	53.9	54.2	48.9	52.0	59.7	50.8	52.8	60.8	62.2	52.2	55.5	49.0	49.7
Socio-economic status**														
low FAS (%)	23.3	14.1	11.3	48.9	12.5	37.3	21.7	9.0	32.3	14.4	9.4	39.1	-	14.2
Medium FAS (%)	46.5	50.2	47.1	41.4	49.3	45.1	51.3	44.0	46.5	44.4	49.2	48.0	-	41.0
high FAS (%)	30.3	35.7	41.7	9.7	38.2	17.6	27.0	47.0	21.2	41.2	41.4	12.9	-	44.8

* Countries: AT = Austria, CH = Switzerland, CZ = Czech Republic, DE = Germany, EL = Greece ES = Spain, FR = France,, HU = Hungry, IE = Ireland; NL = Netherlands, PL = Poland, SE = Sweden, UK = United Kingdom.

** FAS: Family Affluence scale (0–3 = low; 4–5 = medium; 6–7 = high)

### Cooperation, completion and response rates

Table 2 shows the cooperation, completion and response rates for the Kidscreen sample by country. The mean cooperation rate using telephone sampling was 56.5% (range, 42.8% – 76.5%) compared to 89% (range, 69% – 97.2%) for school based sampling (table 1). Completion rates were highest when questionnaires were administered in schools (92–100%), and varied substantially in the mail surveys. The lowest mail survey completion rates were in the UK (44.1 %) and FR (45.3 %); the highest rates were in the NL (97.8%) and DE (77.6 %) (table 2). Response rates were highest in countries that used school sampling and administration during class-time (range from 69.0% in the UK to 91.2% in SE), and lowest in countries which used CATI sampling and mailed surveys (between 18.9% in the UK, and 68% in NL). Countries that used other methods achieved intermediate response rates (59.6% in PL, and 71.5% in CZ). Within both types of sampling and administration strategies, participation and response rates tended to be higher in countries with greater proportions of parents in higher educational categories according to Eurostat data.

**Table 2:** Sampling procedures and data collection methods in countries participating in the KIDSCREEN study: cooperation, completion, and response rates. Countries are ordered by sampling method and alphabetically by abbreviations.

	**Sampling**				**Data collection**				**Response rate**
**Country**	Method	ECC^a^	Coop.^a^	**Coop. rate**	Mode^a^	V.C.^a^	Qfill^a^	**Completion rate**	
		N	n	%		N	n	%	%
**Phone & mail**									
AT. Austria	CATI^a^	4433	2425	**54.7**	Mail	2395	1544	**64.5**	**35.3**
CH. Switzerland	CATI	4349	2423	**55.7**	Mail	2423	1746	**72.1**	**40.2**
DE. Germany	CATI	4642	2430	**52.3**	Mail	2413	1873	**77.6**	**40.6**
ES. Spain	CATI	4009	2052	**51.2**	Mail	1956^b^	924	**47.2**	**24.2**
FR. France	CATI	4222	2459	**58.2**	Mail	2382^b^	1079	**45.3**	**26.4**
NL. The Netherlands	CATI	866	426	**49.2**	Mail	1961	1919	**97.8**	**48.1**^c^
	DIDOC^a^	2549	1949	**76.5**					**74.8**^c^
**Schools**									
EL. Greece	Schools	1656	1192	**72.0**	Schools	1192	1192	**100**	**72.0**
HU. Hungary	Schools	3622	3560	**97.2**	Schools	3560	3297	**92.6**	**90.0**
IE. Ireland	Schools	1534	1265	**82.5**	Schools	1265	1265	**100**	**82.5**
SE. Sweden	Schools	3650	3354	**91.2**	Schools	3354	3354	**100**	**91.2**
**Other**									
UK. United Kingdom	CATI	2517	1079	**42.8**	Mail	1062	468	**44.1**	**18.9**
	Schools	2210	1526	**69.0**	Schools	1526	1526	**100**	**69.0**
PL. Poland	Schools	2915	2411	**82.7**	Mail	2378	1715	**72.1**	**59.6**
CZ. Czech Republic	MPS^a^	2283	1632	**71.5**	Households	1632	1632	**100**	**71.5**

^a ^ECC: adults with eligible children contacted; Coop.: parents who agreed to participate ; Mode: Mode of administration; VC: valid cases or addresses; Qfill: questionnaires filled in by children or adolescents, and sent back; CATI: computer assisted telephone interview; DIDOC: large data services institute in the Netherlands; MPS: Multistage probability sampling for selecting households; ^b^Refusals were confirmed in Spain (n = 205) and France (n = 5) by phone. ^c ^Mean response rate for the NL was 68.0%.

### Participants compared with reference census population data

Table 3 shows the population fractions and fraction ratios by country according to age and sex. On these parameters, the distributions of the KIDSCREEN national samples in each country were quite similar in terms of sex and age to their Eurostat reference populations. Nevertheless, due to the large sample sizes in each country, most of the differences observed were statistically significant (table 2).

**Table 3:** Population fraction (PF) and ratio (PFR) by sex and age in the KIDSCREEN national surveys and the reference population from Eurostat^a^.

Countries ^b^	Sample (n)^c^	Reference population (N) ^a^	Sex	Children (8–11)				Adolescents (12–18)				Adolescent PFR^d,e^
				Sample (%)	Reference population (%) ^a^	Population		Sample (%)	Reference population (%) ^a^	Population		
						Fraction ‰	PFR ^d^			Fraction ‰	PFR ^d^	
Phone & mail												
AT. Austria	1,475	1,044,588	Female	18.8^g^	18.0	1.47	1^h^	35.0^i^	31.0	1.59	1^h^	1.08
			Male	16.3	18.6	1.24	0.84	29.9	32.3	1.31	0.82	1.05
CH. Switzerland e	1,701	675,327	Female	17.9^g^	18.0	2.49	1^h^	35.7^i^	30.6	2.95	1^h^	1.18
			Male	16.2	18.9	2.16	0.87	30.2	32.5	2.34	0.79	1.08
DE. Germany	1,723	10,048,086	Female	17.7	16.9	0.18	1^h^	33.6^i^	31.8	0.18	1^h^	1.01
			Male	17.7	17.8	0.17	0.95	31.0	33.5	0.16	0.87	0.93
ES. Spain	876	4,722,467	Female	16.9	16.4	0.19	1^h^	32.2	32.1	0.19	1^h^	0.97
			Male	19.7	17.5	0.21	1.10	31.2	33.9	0.17	0.92	0.81
FR. France	1,049	8,335,092	Female	18.9	17.3	0.14	1^h^	33.0^i^	31.6	0.13	1^h^	0.95
			Male	18.7	18.1	0.13	0.94	29.5	33.0	0.11	0.86	0.87
NL. Netherlands	1,885	2,150,420	Female	17.7	18.4	0.85	1^h^	33.4^i^	30.4	0.96	1^h^	1.14
			Male	18.2	19.2	0.83	0.98	30.6	31.9	0.84	0.87	1.01
												
Schools												
EL. Greece	1,174	903,105	Female	--	--	--	--	59.7^i^	48.6	1.60	1^h^	--
			Male	--	--	--	--	40.3	51.4	1.02	0.64	--
HU. Hungary	3,237	1,374,408	Female	23.5^g^	17.2	3.21	1^h^	34.9^i^	31.8	2.59	1^h^	0.81
			Male	19.1	18.0	2.50	0.78	22.5	33.0	1.61	0.62	0.64
IE. Ireland	1,240	643,835	Female	13.6	16.5	1.62	1^h^	46.1^i^	32.1	1.16	1^h^	0.87
			Male	12.3	17.3	2.82	1.74	28.0	34	1.75	1.15	0.57
												
Other												
UK. United Kingdom (phone & mail, and schools)	1,877	8,433,968	Female	24.2	18.3	0.30	1^h^	24.2	30.4	0.18	1^h^	0.60
			Male	27.0	19.2	0.31	1.06	24.6	32.1	0.17	0.96	0.55
PL. Poland (schools and mail)	1,715	6,421,859	Female	17.6^g^	15.6	0.30	1^h^	37.2^i^	33.2	0.30	1^h^	1.00
			Male	15.4	16.5	0.25	0.83	29.9	34.7	0.23	0.77	0.92
CZ. Czech Republic (MPS)	1,592	1,426,558	Female	17.7	17.0	1.16	1^h^	31.8	31.5	1.13	1^h^	0.97
			Male	17.3	18.0	1.07	0.92	33.2	33.5	1.11	0.93	1.04

^a ^Census data provided by Eurostat; ^b ^Countries ordered by sampling method and alphabetically; ^c ^Cases with sufficient data to be able to calculate KIDSCREEN reference values. ^d^PFR: population fraction ratio (the 95%CI for the PFR were not included;as all of them were statistically different from the reference category); ^e^Reference category: children; ^f^Census data for Switzerland in the year 2000, in 19 out of 26 cantons where sampling was carried out; ^g^Statistically significant differences by sex within children (p < 0.01) ; ^h ^Reference category: female; ^i ^Statistically significant differences by sex within adolescents (p < 0.01).

The population fraction varied depending on the number of 8–18 year olds in the country, and also on the sample size. In France, the PF was 0.13–0.14/1,000 children, and 0.11–0.13/1,000 adolescents, but rose to 2.50–3.21/1,000 in Hungarian children, and 2.34–2.95/1,000 in Swiss adolescents (table 2).

In the KIDSCREEN sample, girls tended to be over-represented compared to the Eurostat reference population in almost all countries. This was also true across all of the sampling methods. The PF was lower in boys than in girls in almost all countries, except IE, where the male-female PFR was 1.74. In general, in school-based samples females were more likely to participate than males (PFR = 0.64, EL; 0.78–0.62, HU; 0.83–0.77, PL).

The PF was lower in adolescents than in children, except in AT, CH, and DE. In school-based samples, adolescents were less likely to participate than children (PFR = 0.81 in girls, and 0.64 in boys, HU; 0.87 in girls, and 0.57 in boys, IE), ES (0.81 in boys), and UK (0.69 in girls and 0.60 in boys) (table 2).

The external validity was also studied by comparing parents' declared level of education with the Eurostat reference sample. In the majority of countries, parents with a low educational level were less likely to complete the survey materials than parents with an intermediate educational level (e.g. PFR = 0.37 for mothers with a low educational level compared to those with an intermediate level in AT) (table 4). The PFR for parents with a high educational level varied between 0.86 (AT) and 2.20 (FR) in countries which used phone sampling and mail administration, and was over 1.5 in countries which used school sampling.

**Table 4:** Educational level of mothers and fathers participating in KIDSCREEN study compared with EUROSTAT reference population.

Level of education within countries^a,b^		**Mothers**				**Fathers**			
		Sample (%)	Reference population^c^(%)	Population		Sample (%)	Reference population^c^(%)	Population	
				Fraction ‰	PFR (95% CI)^d^			Fraction ‰	PFR (95% CI)^d^
**Phone & mail**									
AT. Austria	Low	11.2^e^	24.3^e^	1.15	0.37 (0.31–0.43)	7.82^e^	13.8^e^	1.55	0.49 (0.40–0.60)
	Medium	74.5	59.1	3.16	1^f^	75.5	65.7	3.15	1^e^
	High	14.2	16.6	2.15	0.86 (0.74–0.99)	16.7	20.5	2.23	0.81 (0.71–0.93)
	Total	n = 1,489	N = 595,200	2.50		n = 1432	N = 523000	2.74	
CH. Switzerland	Low	13.6^e^	15.7^e^	3.10	0.93 (0.80–1.08)	8.18^e^	11.5^e^	2.90	0.98 (0.81–1.19)
	Medium	69.6	74.7	3.33	1^f^	48.7	67.3	2.96	1^f^
	High	16.8	9.6	6.23	1.74 (1.53–1.98)	43.2	21.2	8.36	2.04 (1.86–2.24)
	Total	n = 1,595	N = 446,000	3.58		n = 1492	N = 364000	4.10	
DE. Germany	Low	23.3	20.9	0.33	1.14 (1.01–1.27)	29.4^e^	13.6^e^	0.67	2.79 (2.49–3.13)
	Medium	59.9	60.9	0.29	1^f^	45.2	58.3	0.24	1^f^
	High	16.8	18.2	0.27	0.92 (0.82–1.05)	25.4	28.1	0.28	0.90 (0.81–1.01)
	Total	n = 1,700	N = 5,788,300	0.29		n = 1614	N = 5217200	0.31	
ES. Spain	Low	56.6^e^	63.9^e^	0.23	0.87 (0.73–1.05)	55.4^e^	60.7^e^	0.24	0.90 (0.74–1.08)
	Medium	18.4	18.1	0.27	1^f^	18	17.7	0.27	1^f^
	High	25.0	18.0	0.37	1.39 (1.19–1.61)	26.7	21.7	0.32	1.23 (1.06–1.43)
	Total	n = 844	N = 3,197,800	0.26		n = 784	N = 2986000	0.26	
FR. France	Low	32.0^e^	37.6^e^	0.18	1.63 (1.37–1.93)	38.2^e^	34^e^	0.25	2.66 (2.23–3.17)
	Medium	21.5	41.2	0.11	1^f^	19.2	45.5	0.10	1^f^
	High	46.5	21.2	0.46	2.20 (1.97–2.45)	42.6	20.5	0.47	2.08 (1.85–2.33)
	Total	n = 1,019	N = 4,883,300	0.21		n = 969	N = 4269900	0.23	
NL. Netherlands	Low	20.0^e^	35.5^e^	0.97	0.44 (0.39–0.49)	25.1^e^	29.9^e^	1.59	0.81 (0.72–0.91)
	Medium	57.9	44.9	2.22	1^f^	43.5	41.7	1.97	1^f^
	High	22.0	19.7	1.93	1.12 (1.00–1.25)	31.4	28.5	2.08	1.10 (1.00–1.21)
	Total	n = 1,787	N = 1,037,200	1.72		n = 1747	N = 927300	1.88	
**Schools**									
EL.Greece	Low	36.9^e^	59.5^e^	0.77	0.54 (0.46–0.62)	38.7^e^	53.3^e^	1.62	0.72 (0.61–0.83)
	Medium	36.5	31.5	1.44	1^f^	29.5	29.1	2.26	1^f^
	High	26.7	9.0	3.71	2.97 (2.60–3.40)	31.8	17.6	4.03	1.81 (1.59–2.05)
	Total	n = 1,001	N = 803,000	1.25		n = 974	N = 437000	2.23	
HU. Hungary	Low	29.7^e^	27.3^e^	2.40	1.43 (1.28–1.59)	44^e^	18.3^e^	5.59	5.20 (4.66–5.80)
	Medium	44.0	57.9	1.68	1^f^	31.6	68.2	1.08	1^f^
	High	26.3	14.8	3.94	1.78 (1.61–1.96)	24.4	13.5	4.18	1.80 (1.62–2.00)
	Total	n = 1,901	N = 858,600	2.21		n = 1734	N = 74700	2.32	
**other**									
UK. United Kingdom	Low	13.6^e^	20.4^e^	0.20	0.98 (0.82–1.17)	17.9^e^	14.6^e^	0.41	2.44 (2.05–2.91)
	Medium	38.4	56.5	0.20	1^f^	28.5	56.8	0.17	1^f^
	High	47.9	23.2	0.61	2.07 (1.87–2.28)	53.6	28.6	0.62	1.88 (1.70–2.07)
	Total	n = 1,225	N = 4,163,800	0.29		n = 1119	N = 3362100	0.33	
PL. Poland	Low	32.2^e^	50.1^e^	1.75	0.50 (0.45–0.55)	47.6^e^	61.6^e^	2.42	0.56 (0.50–0.63)
	Medium	49.1	38.0	3.51	1^f^	35.8	25.9	4.34	1^f^
	High	18.8	11.9	4.30	1.58 (1.40–1.78)	16.6	12.5	4.17	1.33 (1.16–1.52)
	Total	n = 1,673	N = 615,000	2.72		n = 1456	N = 464000	3.14	
CZ. Czech Republic	Low	3.7^e^	11.0^e^	0.58	0.36 (0.27–0.46)	2.67^e^	6.12^e^	0.79	0.47 (0.35–0.65)
	Medium	75.1	79.1	1.63	1^f^	73.1	79.4	1.66	1^f^
	High	21.2	10.0	3.65	2.13 (1.89–2.40)	24.3	14.4	3.04	1.68 (1.50–1.88)
	Total	n = 1,558	N = 908,000	1.72		n = 1496	N = 826500	1.81	

^a ^Level of education according to ISCED categories; ^b ^Countries ordered by sampling method and alphabetically; data not available for IE; ^c^Eurostat data for women and men with at least one child from 8–18 years old in the household; ^d ^PFR:population fraction ratio (95% confidence interval); ^e ^Statistically significant difference (p < 0.01); ^f ^Reference category.

### Participants compared with refusals

Table 5 provides data on the characteristics of children and parents in households that refused to participate compared to households who actually participated. Parents who participated were more likely to declare good self-perceived health for both their children and themselves. They were also more likely to be married and have a higher educational level, and they were less likely to live in large cities (except in CH). No statistically significant differences were found between refusals and participants in PL, the only country which used school-based sampling and which also collected data from refusals.

**Table 5:** Characteristics of participants and refusals in the KIDSCREEN study.

Countries^a ^and variables	Participants^b^		Refusals^c^		p
**Phone & mail**	n	(%)	n	(%)	
**AT Austria**	1544		334		
Child's health: good, very good or excellent		98.8		83.1	<0.001
Parent's health: good, very good or excellent		95.5		82.9	<0.001
Parent's marital status: married		87.7		64.3	<0.001
Parent's educational level: high		14.9		33.8	<0.001
medium		74.5		63.5	
Home in big city or suburbs		18.9		18.2	0.815
**CH Switzerland**	1746		496		
Child's health: good, very good or excellent		99.0		98.0	0.099
Parent's health: good, very good or excellent		95.4		90.5	0.000
Parent's marital status: married		85.2		85.8	0.733
Parent's educational level: high		22.1		6.0	<0.001
medium		66.1		50.2	
Home in big city or suburbs		16.5		12.1	0.020
**DE Germany**	1775		367		
Child's health: good, very good or excellent		97.4		98.1	0.580
Parent's health: good, very good or excellent		90.3		90.1	0.922
Parent's marital status: married		84.2		74.9	<0.001
Parent's educational level: high		19.1		29.3	
medium		58.7		65.9	
Home in big city or suburbs		22.8		41.6	<0.001
**ES Spain**	912		360		
Child's health: good, very good or excellent		98.2		96.7	0.092
Parent's health: good, very good or excellent		91.3		82.9	<0.001
Parent's marital status: married		90.6		89.8	0.646
Parent's educational level: high		25.7		20.1	0.114
medium		20.0		20.7	
Home in big city or suburbs		29.0		49.4	<0.001
**FR France**	1079		341		
Child's health: good, very good or excellent		97.5		84.8	<0.001
Parent's health: good, very good or excellent		94.1		82.2	<0.001
Parent's marital status: married		85.7		75.4	<0.001
Parent's educational level: high		45.4		22.0	<0.001
medium					
Home in big city or suburbs		37.5		43.2	0.072
**NL Netherlands**	1919		260		
Child's health: good, very good or excellent		96.8		95.0	0.142
Parent's health: good, very good or excellent		90.4		93.8	0.085
Parent's marital status: married		89.8		90.4	0.824
Parent's educational level: high		24.3		1.4	<0.001
medium		56.0		41.0	
Home in big city or suburbs		46.7		49.2	0.465
**Other**					
**PL Poland**	1715		366		
Child's health: good, very good or excellent		94.3		91.2	0.039
Parent's health: good, very good or excellent		78.7		80.1	0.615
Parent's marital status: married		88.0		90.3	0.235
Mother's educational level: high		18.8		17.1	<0.001
medium		49.1		25.1	
Home in big city or suburbs		40.8		44.1	0.257
**CZ Czech Republic**	1603		344		
Child's health: good, very good or excellent		94.9		89.5	<0.001
Parent's health: good, very good or excellent		88.6			
Parent's marital status: married		84.0		63.7	<0.001
Parent's educational level: high		23.1		19.5	<0.001
medium		73.8		67.7	
Home in big city or suburbs		21.3		23.0	0.516

^a^Data not available for EL, HU, IE, and UK;^b^'Participants' refer to the parent-child pair, i.e. when a parent agreed to participate and survey materials were completed by a parent; ^c ^'Refusals' were the parents who were not willing to participate, but answered a short interview on non-response.

## Discussion

It is agreed that it's important to ensure that reference values for HRQoL instruments are based on representative samples. However, there has been relatively little analysis of aspects such as sampling and administration methods, cooperation and response rates, and the external validity. The fact that the present study was carried out in several different European countries using different sampling and administration methods made it even more important in this case. This sort of analysis also provides information about sampling and questionnaire administration strategies which may be useful for future studies to obtain reference values.

Cooperation and response rates varied considerably by country and by the sampling and administration methods used. As was expected, school-based surveys produced higher cooperation rates, which might be explained by a greater degree of confidence among parents due to the school's involvement from the outset (unsolicited telephone contact vs. an individualized letter from the school)[7]. A further advantage of school-based sampling is that once parental consent to participate was obtained, completion and response rates were almost 100%. On the other hand, one of the main advantages of the centralized CATI method is that it is a relatively efficient way of obtaining broad geographical coverage, which might be more difficult to achieve using school sampling. This needs to be balanced against lower participation and response rates, as well as the fact that phone coverage is limited in some European countries. Whereas the percentage of households with phones was over the 90% in most of the countries included in the present survey [16], it was under 50% in CZ, HU and PL [17], leading to an obvious source of sample bias. For this reason, among others, the survey was carried out using school or household sampling in EL, HU, IE, PL, SE, and CZ.

Using the telephone sampling method, we also observed considerable variation in the cooperation, completion, and response rates across countries, with reasonably good rates in NL and German-speaking countries, but lower rates in ES, FR, and the UK. Similar patterns in response rates have been found in earlier Europe-wide studies in adults, although earlier studies have also noted wide variations in response rates within regions and countries [18].

Differences in participation and response rates between countries using the same sampling and administration procedures could be due to several factors. In particular, it was observed that countries with better-educated populations tended to produce higher response rates, though cultural factors could also play a role. This will have implications regarding expected non-response rates for sample size estimates in different European countries. Other reasons for the variations observed between countries might include differences in the reminder procedures used, or differences in study materials even though considerable effort was made to standardize study materials and follow-up procedures. Similar strategies were also used across countries to increase response rates, such as contacting participants before sending the questionnaire, use of personalized questionnaires and letters, inclusion of stamped addressed envelopes for return of completed questionnaires, and a second telephone contact to non-respondents [19].

Despite differences in sampling and administration methods between countries, the final samples obtained were generally very similar to the reference populations. As is frequently observed in surveys of this type, and as indicated by the population fraction rates, there was a slight tendency for a higher response rate from females compared to males and from children compared to adolescents. This was true across all sampling methods and countries. Likewise, the mothers' educational level in the KIDSCREEN sample was similar to that of the reference sample in most countries, though those in the lowest educational category tended to be underrepresented. Although differences between the KIDSCREEN sample and the Eurostat reference populations were small, they could affect scores on the KIDSCREEN instrument, and therefore the validity of the KIDSCREEN reference values, as HRQoL scores have been shown to differ by age, sex, and level of education [20]. To test the possible effect of these differences on KIDSCREEN global and dimension scores additional analyses using re-sampling methods such as bootstrapping were performed. These indicated no appreciable effect on KIDSCREEN scores (data not shown).

Finally, there were some differences in socio – demographic characteristics between participants and refusals, with non-responders in general having lower levels of education and reporting slightly poorer health. Future studies may need to take this into account by over-sampling these groups to ensure adequate representation in the final sample.

The study had some limitations. In particular, the fact that the results cannot be generalized beyond the countries studied and the fact that there may have been some confounding between the countries and the different sampling approaches. Whereas the former limits the study's external validity, the latter might affect the internal validity of the conclusions. Future studies could focus on applying and comparing different strategies within the same country. The results could also be due to psychological and social factors that were not included in the present analysis [21], although the literature indicates that the main factors associated with non-response were included, i.e. age, sex and socioeconomic or educational level[8,9]. Finally, although we made considerable efforts to ensure that standardized procedures were used for follow-up, there may have been some differences between countries that we were unable to control. Any such differences could affect the study conclusions, particularly as aspects such as reminder letters and follow-up phone calls have been shown to have a considerable impact on response rates [18,22].

## Conclusion

We found that school-based surveys provided the highest cooperation and response rates, although they did not necessarily provide the most representative samples, at least in terms of age and sex. This approach also requires sophisticated sampling strategies in order to avoid biases, and gaining access to schools may not always be easy. Centralized phone sampling and mail administration led to slightly lower cooperation rates, although this was variable between countries. This approach also led to a lower rate of participation amongst those in lower educational categories, although the distribution by age and sex was more representative than that achieved with school sampling. Finally, multistage random sampling of communities and households achieved a well-balanced age-sex, and urban-rural survey, but at a higher cost than other methods. The most appropriate method to use will likely depend on researchers' available resources, telephone coverage in target countries, and the need for representativeness on given variables. This study has also indicated some areas in which oversampling and additional follow-up strategies may be required. Finally, the results suggest that the KIDSCREEN survey has achieved a sufficient degree of representativeness to provide reference population values, which will improve the interpretability of the KIDSCREEN questionnaire.

## Competing interests

The author(s) declare that they have no competing interests.

## Authors' contributions

URS, WD, JA, and LR conceived the KIDSCREEN study, and participated in its design and coordination, and helped to draft the manuscript. SB, UvR, and LR designed the collection of data, and the analysis of representativeness. SB, CT, and ME made the management and analysis of data. SB wrote the first draft of the manuscript. CB, MH, and the rest of authors made substantial contributions to the interpretation of data, revised critically all the previous versions of the manuscript, and approved its final version (see acknowledgements).

## Pre-publication history

The pre-publication history for this paper can be accessed here:


